# Mental health in primary health care in a rural district of Cambodia: a situational analysis

**DOI:** 10.1186/s13033-018-0185-3

**Published:** 2018-01-24

**Authors:** Sofia Olofsson, Miguel San Sebastian, Bhoomikumar Jegannathan

**Affiliations:** 1 Epidemiology and Global Health, Department of Public Health and Clinical Medicine, Umeå University, Umeå, Sweden; 2 Centre for Child and Adolescent Mental Health, Takhmau, Cambodia

**Keywords:** Mental health, Primary health care, Integration, Rural, Cambodia

## Abstract

**Background:**

While mental and substance use disorders are common worldwide, the treatment gap is enormous in low and middle income countries. Primary health care is considered to be the most important way for people to get mental health care. Cambodia is a country with a long history of war and has poor mental health and limited resources for care. The aim of this study was to conduct a situational analysis of the mental health services in the rural district of Lvea Em, Kandal Province, Cambodia.

**Methods:**

A cross-sectional situational analysis was done to understand the mental health situation in Lvea Em District comparing it with the national one. The Programme for improving mental health care (PRIME) tool was used to collect systematic information about mental health care from 14 key informants in Cambodia. In addition, a separate questionnaire based on the PRIME tool was developed for the district health care centres (12 respondents). Ethical approval was obtained from the National Ethics Committee for Health Research in Cambodia.

**Results:**

Mental health care is limited both in Lvea Em District and the country. Though national documents containing guidelines for mental health care exist, the resources available and health care infrastructure are below what is recommended. There is no budget allocated for mental health in the district; there are no mental health specialists and the mental health training of health care workers is insufficient. Based on the limited knowledge from the respondents in the district, mental health disorders do exist but no documentation of these patients is available. Respondents discussed how community aspects such as culture, history and religion were related to mental health. Though there have been improvements in understanding mental health, discrimination and abuse against people with mental health disorders seems still to be present.

**Conclusions:**

There are very limited mental health care services with hardly any budget allocated to them in Lvea Em District and Cambodia overall. There is dire need for scaling up and integrating mental health into primary health care to improve the population’s access to and quality service of Cambodian mental care.

**Electronic supplementary material:**

The online version of this article (10.1186/s13033-018-0185-3) contains supplementary material, which is available to authorized users.

## Background

The World Health Organization (WHO) describes mental health as an integral and essential part of health and that there is no health without mental health [1]. It has been reported that mental and substance use disorders are responsible for 6.77% of the global disability-adjusted life years (DALYs) [2] and the estimation is that 80% of people with serious mental disorders living in low and middle income countries do not receive sufficient mental health services [3]. Acknowledging the burden of mental health problems, WHO has developed the Mental Health Gap Action programme (mhGAP) to scale up services for mental, neurological and substance use disorders at the primary care level [4] and the Mental Health Action plan 2013–2020 to expand services for mental health in low resource settings [5].

Cambodia is a lower middle-income country in Southeast Asia with an estimated population of 15.6 million [6]. The majority of the people are Buddhists and the main language is Khmer. Cambodia’s total landmass is 181,035 sq. km [7] and there are 24 provinces and 185 districts [8]. Considering the fragile states index, Cambodia is listed as number 46 out of 178 countries [9].

Approximately 85% of the Cambodian population lives in rural communities and the mental health facilities are located in urban centres [10]. Today Cambodia has 1049 primary health care (PHC) centres that cover 10,000–20,000 people each. However, in a report from 2010 only 43% of the PHC services provided the full minimum package of services required by the government [11]. PHC is considered to be the best way for people to receive mental health care; people can access the service closer to their homes, and in addition, stigma and discrimination are minimized [12]. The evidence suggests that mental health care can be delivered effectively in PHC settings with the help of community-based programmes and task-shifting approaches. Basic training in mental health and appropriate supervision by mental health specialists can contribute to non-specialist health professionals’ ability to detect, diagnose and treat patients with mental health disorders [13], reducing the number of unnecessary investigations and inappropriate and non-specific treatments. It is a complex task of integrating mental health care into PHC but evidence has shown that community-based services are more effective and cost effective than hospitals [4].

War affects the health and wellbeing of a country in a fatal way, and mental health is one of the most impacted areas. Cambodia has a long history of war and violence where the civil war in the 1960s and the Khmer Rouge period in the 1970s are the most recent ones [14]. During the Khmer Rouge period, it has been estimated that up to two million Cambodians died due to execution, overwork, starvation and disease. Much of the civil infrastructure, including the judicial and health systems, were destroyed. The Khmer Rouge period together with widespread poverty, high rates of violence against women and a precarious human rights situation are contributory causes to the poor mental health in Cambodia [10].

The aim of this study was to make a situational analysis of the mental health services in Lvea Em District, Kandal Province, with a health system perspective in order to facilitate the planning of mental health services by governmental policy makers.

## Methods

### Setting

Lvea Em District in Kandal Province is a flood plain on the north bank of the Mekong River located 70 km to the east of Phnom Penh, accessible only by boat. The district is divided into 15 communes and 43 villages. Lvea Em is a rural area and, therefore, represents how the majority of Cambodia’s citizens live [15]. There are 11 health care centres and the population is around 82,000. To the best of our knowledge, there is no existing mental health clinic in the district and no research has been done about mental health.

The district was selected because of its location close to Phnom Penh, it is representative of a rural context and there was already an established contact due to community programmes implemented by the Centre for Child and Adolescent Mental Health (CCAMH) over the past decade. CCAMH is the only centre in Cambodia providing specialised mental health care for children and adolescents. The centre was established in 1991 and is a collaborative project between the Ministry of Health (MoH), the Royal Government of Cambodia and Caritas Cambodia, an international NGO. This research was performed in collaboration with the team at CCAMH.

### Study design and the situational analysis tool

**Fig. 1:**
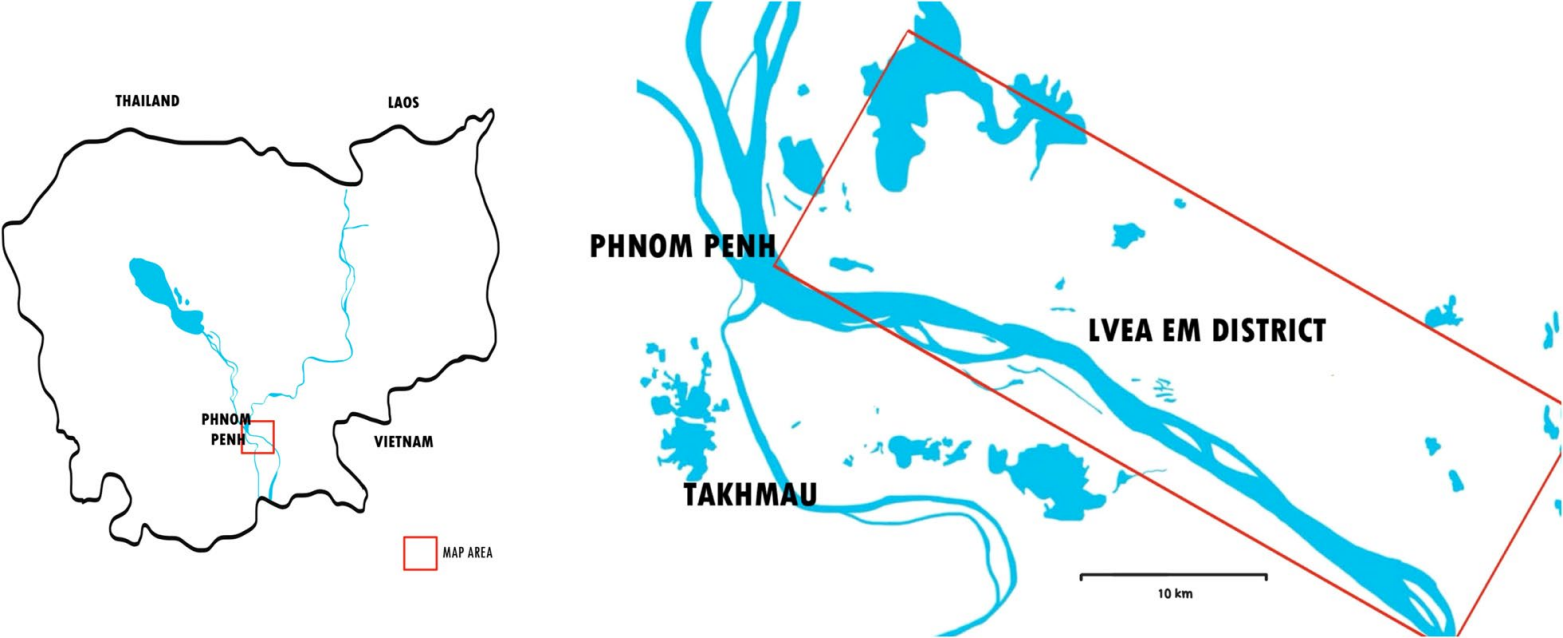
Map of Cambodia and Lvea Em District

A cross-sectional situational analysis was done for this study. To understand how the current mental health situation is in Cambodia and Lvea Em District, the PRIME tool was applied. PRIME, Program for improving mental health care, is a consortium of research institutions and ministries of health in Ethiopia, India, Nepal, South Africa and Uganda, with partners in the UK and the WHO. This is a 6 year programme launched in 2011 with aims to improve the coverage of treatment for priority mental disorders by implementing and evaluating the WHO’s mental health Gap Action Programme (mhGAP) guidelines [16]. The PRIME tool consists of seven sections [17]:

- Section Ι (*Relevant context; 39 items*): covers environmental, population, economy, health (including reproductive health and HIV) and social interactions.
- Section ΙΙ (*Mental health politics, policies and plans; 15 items)*: collects information on national and local political support for mental health care, including budgets, policies, plans, legislation and welfare benefits, and details of the specialist mental health workforce at the national level.
- Section ΙΙΙ *(Mental health treatment coverage; 19 items*): searches for the prevalence of mental, neurological, and substance use (MNS) disorders, numbers receiving care and estimated treatment coverage.
- Section ΙV (*District level health services; 62 items*): gathers information on general health services including the available cadres of health professionals at each level in the system, specialist mental health services, with particular reference to elements of specialist mental health care needed to support PHC-based mental health care and deliver of the mhGAP packages [18, 19], the extent and nature of mental health care delivered in primary care and health system structures to support integration of mental health care into PHC.
- Section V (*Community; 17 items*): includes sociocultural aspects, relevant non-health sector organizations and awareness-raising activities.
- Section VΙ (*Monitoring and evaluation; 4 items*): focuses on health information systems, and monitoring and evaluation of services.
- Section VΙΙ (*Key stakeholders; 1 item*): collects the key stakeholders for mental health in the country and the district (Fig. 1).

### Data collection and participants

Data for section Ι was collected from written reports and national level websites and from key actors in Lvea Em District. Information about the national and provincial level for sections ΙΙ to VΙΙ was collected through individual interviews with key persons (directors, experts on mental health in Cambodia and clinician) in both the general health sector, mental health sector and from NGOs. Data from the district level was collected by personal interviews with the Representative of Health (sections Ι to VΙ), the Representative of Administration in Lvea Em District (section Ι) and by applying a questionnaire to the directors (who also worked as nurses or midwives) of all the 11 health care centres and from one medical doctor at the referral district hospital (sections ΙΙΙ to V). Additional information for completion of the situation analysis was drawn from web-based publications for section Ι [6, 7, 20] and governmental reports for section ΙΙ [22–25].

Individual interviews were done with 14 respondents (Table 1), conducted by the first author. English was possible in nine of the interviews, and for the other five, one medical doctor from CCAMH and one member of the research team were involved in the translation from Khmer to English. Most of the interviews were held at the working places of the respondents, the other ones at suitable meeting points for the respondents. All interviews were held in, or in the region around, Phnom Penh. All interviews were based on the PRIME tool and the topic guide varied due to the profession of the respondent. Due to the extent of the PRIME tool, and limited time for each interview, all questions were not relevant for all respondents. The estimated mean time for each interview was 90 min.

**Table 1:** Respondents participating in the face-to-face interviews

Respondents, title	Sections
District level	
Representative, Health, Lvea Em District	Ι–V
Representative, Administration, Lvea Em District	Ι
Midwife, Lvea Em District	ΙΙ–V
Provincial level	
Representative, Provincial Health Department, Kandal Province	ΙΙ–VΙ
Representative, Chey Choum Nean Hospital	VΙ
National level	
Professor and Psychiatrist, Phnom Penh	ΙΙ–VΙΙ
Representative, Department of hospital, Phnom Penh	ΙΙ, ΙΙΙ, V, VΙ
Psychiatrist, Phnom Penh	ΙΙ–VΙ
Representative, Department of Mental Health and Substance Abuse	ΙΙ–VΙ
Psychiatrist, Phnom Penh	ΙΙ–VΙ
NGO	
Psychiatrist, Transcultural Psychosocial Organization (TPO) Cambodia	ΙΙ, ΙV–VΙΙ
Psychologist and Community Programme Manager	Ι–ΙΙΙ
Pediatrician	Ι–VΙ
Psychiatrist	ΙV

The PRIME tool was pre-tested and adapted by the authors together with one of the health care workers in Lvea Em District (see Additional file 1: Appendix S1). The aim of the adaption was to create a shorter questionnaire due to the PRIME tool extent and the limited time for the data collection and to include information about the children’s mental health situation. The final questionnaire was adapted for use at the health care centers in the district and included items from sections ΙΙΙ, ΙV and V and two additional questions about children’s mental health were added. The disorders chosen for section ΙΙΙ were a combination from the existing PRIME tool, disorders mentioned in the Ministry of Health (MoH) Minimum Package of Activities (MPA) and disorders relevant for the work of CCAMH [23]. The questionnaire was translated to Khmer by two members from the research team (one was also involved in interpreting the interviews) and one member translated the answers from the questionnaires.

The situational analysis tool was applied between October and November 2015 by the first author with the assistance of research members at CCAMH.

### Data analysis

Five of the English face-to-face interviews were recorded and transcribed, and notes were taken from the other interviews. All data was compiled in two different excel files and one word document and presented both in a quantitative and qualitative form. Descriptive statistical analysis was conducted on the quantitative data. In this study, key themes from the original PRIME study were the frame for the qualitative interviews and for the structure of this article [25]. Data were analysed, inspired by the framework analysis approach [26].

The district level information was analysed in the context of data gathered at the provincial and national levels.

### Ethics statement

Ethical approval for the research was obtained from the National Ethics Committee for Health Research in Cambodia. Necessary permission to implement the research in Lvea Em District was approved from the Director of Health Operational District, Lvea Em District and the Director of Provincial Health Department, Kandal Province.

Informed consent was obtained from all respondents for the adapted questionnaire in Lvea Em District (12 total). All 14 individuals interviewed signed the form for informed consent. All participation was voluntary and all respondents approved their participation in the study. The authors have the approval to use the respondent’s names in the report (except one) but have chosen to not use their names directly. Preliminary results were presented to the district level staff and the final report was sent to the concerned authorities at all levels.

Approval to use the PRIME Situational Analysis Tool was obtained from PRIME, University of Cape Town, South Africa.

## Results

The results are divided according to the sections in the PRIME tool combining both information from the district and the national level.

### Section Ι: The context of Lvea Em District and Cambodia

There were similar contextual aspects similar in both Lvea Em District and Cambodia, such as the ethnic and religious characteristics (Table 2). While the educational level was better in Lvea Em compared to the national average, the life expectancy was slightly lower. The percentage of households with electricity were nearly three times higher in Lvea Em compared to country average; in contrast, the access to a clean water supply was better in Cambodia as a whole.

**Table 2:** Relevant sociodemographic variables comparing Lvea Em District and Cambodia

	Lvea Em District	Cambodia
Geography	260 sq. km	181,035 sq. km
Administrative unit	Council included several offices and departments	Constitutional monarchy, Parliamentary representative democracy
Administrative unit health care	Head, Director, of Operational District (OD). Committee member for women and child sector	Ministry of health, Provincial health department
Population	82,888	15,577,899
Population density (persons/km^2^)	318.8	86.0
% rural	100%	79%
Ethnicity	Khmer 98%, Vietnamese 2%	Khmer 97.6%, Cham 1.2%, Chinese 0.1% Vietnamese 0.1%, other 0.9%
Language	Khmer	Khmer
Religion	Predominantly Buddhism	Buddhism 96.9%, Muslim 1.9%, Christian 0.4%, other 0.8%
Literacy	80%	73.9%
% households with functioning latrine	50%	42.4%
% households with clean water supply	40%^a^	75.5%^b^
% households with electricity supply	90%	31.1%
Major economic activities	Agriculture, fishing	Agriculture, apparel industry, tourism
Life expectancy	65–70 years	71.7 years
Total fertility rate	2.6	2.8
HIV prevalence	0.2%	0.6%

^a^Need to be boiled before drinking, can be used directly for cooking and bath

^b^Improved water source

### Section ΙΙ: Mental health policies and plans

The Ministry of Health in Cambodia is responsible for determining the policies and direction of the health system in Cambodia including its higher education component. The health strategic plan (HSP) for the years 2008–2015 was written by the MoH with the purpose of addressing health needs of the population there mental health was included as part of the non-communicable diseases control plan. Three of the key components in the strategy for mental health were:

- To develop and expand specialised service for mental health, including substance abuse, through promotion and prevention activities to all provinces; extension of psychiatric inpatient and outpatient service to all complementary package of activities (CPA) level 3; improvement of psychiatric coverage to level 1 and 2 CPA hospitals; and establish new, additional, regional psychosocial rehabilitation centres at selected level 3 hospitals;
- To set up an mental health database for research, performance monitoring and accountability;
- To develop an mental health policy to ensure harmonisation and orderly development of services for the mentally ill, and develop a national mental health law to regulate the practice of psychiatry, psychiatric nursing and allied professionals and protect the patients and professionals.

In addition, Cambodia has a project named the Second Health Sector Support Project (HSSP2). The function of this project is to give support to the Health Strategic Plan and improve access to (and utilization of) effective and efficient health services.

In the MPA for Health Centre Development 2008–2015, mental health was included in the Non-communicable Disease Services section. Two of the overall goals of the MPA were to promote awareness of non-communicable diseases and risk factors and to provide care-treatment services for non-communicable diseases. The CPA was the corresponding national guidelines for referral hospitals, in the CPA guideline from 2006 to 2010 mental health service was included.

There is also a national Mental Health and Substance Misuses Strategic (MHSM) plan whose goal is to reduce the burden of disease associated with mental health illness and substance misuse, as well as other mental health related problems. One of the guiding principles in this plan is to ensure that the provision of mental health was integrated in the MPA and CPA and mainstreamed into relevant services. One of the development objectives in the mental health plan describes how to develop legislation and regulations for strengthening mental health and substance misuse service delivery.

From the interviews, an existing political commitment for mental health services in the district is described but actors from the district noted that implementation was lacking.

A respondent from the national level stated that the Department of Mental Health and Substance Abuse was recently formed (autumn 2014) and more experience was needed. Four of ten respondents were aware of the existence of the ‘mental health and substance misuse strategic plan 2011–2015’, two respondents described another mental health plan for 2002–2022.

One clinician and one NGO representative described the implementation of the mental health plan and the mental health situation in Cambodia in this way:

*‘They have a plan but they don’t work with it’.*

*‘Not enough resources for mental health in Cambodia. The problem is that there is not enough resources’.*

*‘Mental health use to be neglected by health leaders’.*

In the interviews, it was reported that the mental health budget in Cambodia was less than one per cent of the total health budget but none of the respondents could give an accurate number. In a report from 2012 the mental health budget in Cambodia was only 0.01% [27]. There was no budget allocated for mental health in Lvea Em District.

All respondents admitted the lack of legislation for mental health care in Cambodia. Respondents from the national level reported that if a patient with mental health disorders refused necessary treatment it was commonly up to the family to decide if the patient would receive the treatment or not due to the fact that no legislation existed.

A summary comparing mental health policies and plans in Lvea Em District and Cambodia can be found in Table 3.

**Table 3:** Mental health policies and plans in Lvea Em District and Cambodia

	Lvea Em District	Cambodia
Political commitment for mental health services	Yes	Yes
Mental health especially mentioned in general health policy	Yes	Yes
Mental health budget as % of total health budget	0	< 1%
Mental health policy	No	Unclear
Mental health plan	No	Yes
Mental health legislation	No	No

### Section ΙΙΙ: Mental health treatment coverage

None of the interviewed in this study could report the prevalence of mental health disorders in Cambodia. A respondent from the national level stressed:

*‘There is a major need to do a mental health prevalence study. In my estimation that’s the major research needed for now’.*

A key actor in Lvea Em District acknowledged that there were no existing data for the prevalence of main mental disorders at a local level. For information on mental disorders in children and adolescents, CCAMH was mentioned as a possible data source.

No reporting system of mental health disorders exists in the health care centres. The head of the health centres mentioned that they were aware that mental health patients attended the health facilities but no records were kept. All disorders asked about did exist in the district. As one health centre head expressed about the numbers of patients with mental health disorders:

*‘The numbers are not exactly, roughly. The data is based on my own experiences and knowledge’.*

One of the heads explained that when they meet patients with mental health disorders, either at the health care centre or in the village, the patients had usually already been diagnosed by the Khmer Soviet Friendship Hospital (KSFH) in Phnom Penh and had medicine.

One respondent from the national level reported that suicide was one of the specific mental health indicators in Cambodia and that the estimated prevalence of suicide in the county was around 44 per 100,000 persons (the world average was 17 per 100,000). Other indicators that were described were the prevalence of depression, anxiety, psychotic cases and alcohol use and aspects in the society such as violence and poverty.

### Section ΙV: Availability of mental health services

The representative of health in Lvea Em and primary health care staff reported that 11 non-physician based health care centres in Lvea Em District offered services such as general health check-ups for adults and children, maternal care and vaccinations. Each health care centre covers 2–7 villages and the number of patients with physical problems varies from 2622 to 14,177 patients annually. There is one referral hospital in the district with 25 beds for in-patient care, and 9 more beds for in-patient care are located in one of the health care centres. There are six doctors working in the district, in addition to one medical assistant, one dentist, 15 degree nurses, 12 diploma nurses, 34 midwifes (20 secondary and 14 primary) and one physiotherapist. The average distance for the patients to travel to the closest general in-patient facility is 15.2 km. According to respondents from the district there is one referral hospital in the city Kandal in Kandal Province with extensive care.

#### Specialist mental health services

There are no mental health specialists in Lvea Em District. The closest in-patient facility for mental health care is KSFH, 40 km from the referral hospital in Lvea Em District; only 2 of 11 participants in the districts were aware of this. One psychiatrist in Phnom Penh said that 10 psychiatrists worked in KSFH, 10 in-patient beds were available and the hospital was open 24 h a day all week. Only one of 10 respondents from the health care centres reported KSFH as the closest in-patient facility.

Health care workers said that approximately 10 patients per month were referred from health centres in the district to specialist mental health services. There seemed to be a referral procedure from PHC centres to secondary or tertiary care but there are uncertainties about it. For instance, participants explained that a patient who needed mental health care usually had an appointment first in the health care centre and thereafter was brought to a hospital outside the district, either by their family or by ambulance. A referral letter might have been written. At the national level the need for a referral system has been recognised, both from the PHC centres to secondary or tertiary care and the vice versa, but currently, the referral system is not working systematically. A participant from the national level expressed it in this way:

*‘… in the health plan we will hand over the case to the place nearby the patients home but we can not assure at this time that the health centre close to the patient have mental health service…. No one refers back at this time because we are not sure where the place, where to hand over, where to continue to take care of the patient…. No one refer back because don’t know resources at primary health care centre’.*

Another respondent from the national level acknowledged that a system about the care of patients, including a referral system, might be developed in the future.

#### Education, knowledge and service training

The heads of the health care centres stressed that no pre-service training had been provided in mental health for PHC workers. Around one-third of the nurses have had training in mental health care at least 2 days in the last year, provided by CCAMH. The nurses had been trained in child mental health and development but also in aspects related to maternal care. One respondent also commented that the medical doctors had very limited training in mental health during their education.

Psychiatry has recently been included in the medical programme for undergraduate students, and 30 theoretical hours per year was allocated for mental health during year 4–6. One psychiatrist (former medical and psychiatrist student in Phnom Penh) explained that medical students now might have an internship in psychiatry during their education but not necessarily everyone has that possibility. To become a specialist in psychiatry in Cambodia, 3 years of education are necessary and in 2015, only four psychiatrist residents graduated.

There is no existing education for psychiatry nurses in Cambodia; the only psychiatry nurses in the country were educated during a training programme by a Norwegian university in the late 1990s to the beginning 2000s. No education for psychiatry nurses has been maintained in the country after the support from abroad ended.

An experienced psychiatrist in Cambodia expressed the importance of a patient-centred approach and that a patient might have many problems when coming for consultation. The respondent mentioned the great value in understanding the involvement of neurology in psychiatry and the need for cooperation between medical doctors and psychologists, which is currently absent. It was stressed that overall there is a lack of understanding between somatic and psychiatric disorders and therefore, there is a lot of focus on physical symptoms both from the caregivers and the patients themselves. One clinical psychiatrist explained that some patients did not talk about their mental illness and that they described their mental health symptoms only with physical symptoms; for instance, the patient could say,

*‘I have stomach problem, I have heart problem.’*

#### Mental health treatments

The essential drug lists for Lvea Em District and Cambodia are presented in Table 4. The heads of the health care centres said that no psychotropic drugs were available in the district, though the district director reported that Diazepam and Phenobarbital should be present.

**Table 4:** Essential drug list

	Lvea Em District	Cambodia
Anti-psychotics (po)	0	Haloperidol, chlorpromazine, perphenazine
Anti-psychotic depot	0	Haldol Decanoate
Anti-depressants	0	Amitriptiline, clomipramine imipramine, nortriptilin, fluoxetine
Anxiolytics	Diazepam	Diazepam, alprazolam
Mood-stabilisers	0	Carbamazepine, lithium
Anti-epileptics	Phenobarbital	Phenobarbital, carbamazepine, phenytoin
Anti-parkinson	0	Trihexyphenidyl

*Po* per os, oral administration

The regulations about prescriptions for psychotropic drugs are not unified, with several respondents describing different guidelines:

*‘No restriction about prescribing. Everybody are allowed to prescribe, nurses prescribe psychotropic medication … the concerns around prescription is usually around the side effects’. (Representative, national level)*

*‘Only doctors, for mental health only psychiatrist. Nurses can’t prescribe. General doctor can prescribe general medicine but not psychiatry medicine… maybe another rule or another mechanism for general doctor in primary health care’. (Psychiatrist)*

*‘Depend on age and where you are. General doctors are allowed, if they are trained they prescribe, if not trained not allowed’. (Psychiatrist)*

The costs of psychotropic drugs differ depending on the patient’s economic situation. If the patient is poor they might have access to an equity fund covering the consultation fee and medications.

The psychosocial therapies that are available in the district are reduced to the form of advice. For instance, these expressions were used by the health workers when referring to the therapies:

*‘Don’t worry. Take it easy. Don’t be stressed. Don’t drug abuse. Don’t drink alcohol’.*

#### Mental health care in primary health care

From the questionnaires and interviews the very limited access to mental health services at the district level is evident (see Tables 5, 6). Health care staff reported that no mental health services were provided in the district but if a patient with mental health came to the health service, health workers tried to take care of the patient. Minor mental health problems such as anxiety and sleep problems might be treated since drugs should be present (see Table 5).

**Table 5:** List of resources available in Cambodia and Lvea Em District

Service	Lvea Em District	Cambodia
In-patient mental health facilities	No	Yes
Nearest mental health specialist in-patient facility	Khmer Soviet Friendship Hospital, 40 km from referral hospital Lvea Em District	–
In-patient facility for alcohol abuse	No	–
Out-patient mental health facilities	No	Yes
Alcohol detoxification	No (yes)	–
Psychological therapies	Limited	No/limited
Supported housing for patients with mental health disorders	No	No
Rehabilitation for patients with mental health disorders	No	No
Mental health care for perinatal women	No	Yes
Mental health care for patients with HIV/AIDS	No	Yes
Human resources		
Psychiatrist	0	60
Psychiatric nurse	0	40
Psychologist	0	Unclear data
Mental health social worker	0	No data
Occupational therapists	0	0

**Table 6:** Systems to support mental health care in primary health care

	Lvea Em District	Cambodia
Mental health coordinator	No	No
Supervision system	No	No
Mental health detection or screening tools	No	Some places have; NGOs might use, for example, Hopkins Symptom Checklist
Guidelines and treatment protocols for mental health care	No	No
Training manuals on mental disorders	No	No
Contact between PHC workers and mental health professionals	Limited	
Community workers	Yes	–
Volunteers or faith-based organizations	Yes	Volunteers in some places
Detection of patients with mental health disorders who drop out of care	No	–
Any existing data or reports of implementing mental health into PHC	No	–
Awareness about mhGAP	No	Limited awareness

It was not clear if alcohol detoxification was present in the district or not; while the representative of health mentioned that it was not offered, three of the health care centres affirmed the service’s availability in their centre.

No screening tools, guidelines or treatment protocols for mental health are available in the district. There is no knowledge about mhGAP either at district or at provincial level. Lvea Em District has community workers and volunteers who are a link between the population and the PHC centres and help to detect patients who dropped out of care, though it was unclear if this included patients with mental health disorders.

### Section V: Community

Psychiatrists at the national level and staff from Lvea Em District explained that there was evidence on that the population is help seeking for mental health disorders. In Lvea Em District, as well as in the whole country, stigma and discrimination against patients with mental health disorders are still present. One respondent from the district observed that:

*‘For some family and people they have discrimination. People in community don’t talk with these families and patients’.*

A respondent from the national Transcultural Psychosocial Organization (TPO) explained that abuse of patients with mental health disorders still occured, which has also been observed in Lvea Em District. According to health care staff in the district, the understanding about mental health disorders and compassion from the patient’s families has increased in recent years. Awareness raising, anti-stigma and prevention activities for mental health disorders are limited in the country and the district, though some activities have been done and are on-going mostly by NGOs where, for example, TPO have started to educate families and communities on mental health. Clinical psychiatrists explained that radio and newspapers have been used to inform the population about mental health disorders.

Most of the respondents at the district and national levels mentioned that cultural explanations for mental health disorders such as ghost possessions, spirits inside the body and disgracing of ancestors were common. Traditional healer and mediums are still present in Cambodia and some people might have visited them before they visited a medical doctor for their mental health disorder. Although there are traditional healers in Lvea Em District, only one of the PHC centres reported that they have interacted with a traditional or religious healer in the last year.

One psychiatrist proposed that one way to reach people with mental health disorders could be to train the traditional healers and mediums in mental health since these people are often the frontline workers in the community. Another way to reach patients with mental health disorders could be to increase the access to the health care system, as expressed by respondents at the provincial level:

*‘Regarding for roads for example, important to give access to mental health, also a part of the work for improving mental health care. Other development works also have to be done, for example schools far away; build schools and primary health care centres closer to the people, that is also a way to solve the mental health problem’. (Representative, provincial level)*

*‘If you start to get a healthy community you start to get less mental illness’. (Psychiatrist, national level)*

One participant from the national level mentioned that history and religion were issues related to mental health. Knowledge about the history of the country might help to identify traumatic events or episodes experienced by the patients. One idea concerning religion, expressed in an interview, is to include Buddhism in mental health care in Cambodia because of the religion’s significance for the country and a way to understand the patient better.

### Section VΙ: Monitoring and evaluation

Respondents from both the national and provincial levels said that there was an existing general health information system in Cambodia but not a separate one for mental health care, though HIV and TB had their own reporting systems. Furthermore, there is no monitoring evaluation system for quality of mental health care in PHC in Cambodia. One of the psychiatrists in Phnom Penh expressed that mental health care was included in the information system but it was not comprehensive yet. According to respondents at the national level, there is a need for a system to identify patients since today there is no unified code for the patients in Cambodia.

### Section VΙΙ: Key stakeholders

Two respondents from the national and provincial levels reported CCAMH, TPO and Social Services in Cambodia (SSC) as key stakeholders for the work with mental health in the country.

## Discussion

These results provide systematic information about the situation of mental health care in Lvea Em District and Cambodia. No other studies about mental health have been done in Lvea Em District; therefore, this baseline is an important tool for the planning of further interventions for improvement and implementation of mental health care into the PHC with community involvement both at the district level and nationally.

### The context of Lvea Em District and Cambodia

Despite Lvea Em’s close location to the capital Phnom Penh, it is defined as a rural area. However, the proximity to Phnom Penh may influence the living conditions, the disease burden and the access to health care making its representability as to other rural districts uncertain. In the case of studying the mental health situation, the adoption is that the district as eventually more developed then other districts might be less significant.

### Mental health policies and plans

There are both similarities and differences between Lvea Em District and Cambodia as a whole according to politics, policies and plans for mental health. The limited implementation of mental health care into PHC has resulted in a minor focus on mental health reflected in the low resources and knowledge in the district. Reports from WHO have also indicated that the mental health plan was only partly implemented in the country [28]. Although Cambodia has a budget for mental health, it is far below sufficient (0.01%) when compared to high-income countries where it is usually around 5.1% of the total health care budget or even in other low-income countries where it can be around 0.5% [3]. No legislation for mental health has been created even though it had been described as a development objective in the mental health plan. By comparing the background information with data and quotes from the respondents, it seems like the plans, guidelines and goals were far from being fulfilled.

### Mental health treatment coverage

The absence of adequate data on the prevalence of mental health disorders in the district and in the country contributes to the lack of knowledge about the needs, although the high prevalence of suicide may be a proxy indicator of the poor mental health status in Cambodia. The results from the district suggest that mental health disorders probably exist but require further investigation. The lack of epidemiological data has contributed to the marginalisation of mental health in Cambodia; also, a reduced interest about prevalence for mental health disorders compared to somatic disorders has been observed [10].

### Availability of mental health services

The limited resources of mental health specialists and mental health care both in the district and in the country, along with the lack of knowledge about its availability, are huge challenges for the improvement of the mental health care system. The 60 psychiatrists in Cambodia corresponds to one psychiatrist per nearly 260,000 inhabitants, while in Sweden the corresponding number is one psychiatrist per nearly 4400 inhabitants [29, 30].

The lack of a systematic referral system, between the specialist mental health services and the PHC might contribute to the reduced knowledge about the availability of specialist mental health service in the district. With no referral system the quality of care for patients with mental health disorders is impaired, both for initial care from a specialist but also for the follow-up in the PHC. A functioning and reliable referral system can improve the value of mental health care and also strengthen the collaboration between the specialist mental health services and the PHC.

One of the most important issues seen from the results was the low amount of human resources in mental health with very limited education for specialists in mental health. With a low number of graduates specialising in mental health annually, and an existing aging group of specialists, Cambodia’s human resources in mental health might become even more limited in the future. The lack of human resources can also delay the work of implementing mental health into PHC.

The PHC workers in Lvea Em District do not have any pre-service training and very limited in-service training for mental health disorders and mental health care, nor is there a specialist in mental health care or any screening tools or guidelines for how to diagnose mental health disorders and how to give treatments. Most of the mental, neurological and substance use disorders can be managed by non-specialist health care providers and therefore, the mhGAP Intervention Guide was developed for use in non-specialised health-care settings and for the integration of mental health care into PHC. The intervention guide includes guidance on evidence-based interventions to identify and manage several mental health conditions [18] and has potential to be implemented in the daily work in Lvea Em District.

The heads of the health care centres and the representative of health in Lvea Em District gave different answers about the availability of psychotropic drugs in the district. There were also different reports from the participants about where the closest inpatient mental health facility was located and the presence of alcohol detoxification services in the district. The reason for this is not clear but some explanations could be that many people are unaware, there is limited communication and there is misunderstanding among the health staff.

### Community

From the respondents’ point of view, there seems to have been an improved awareness and understanding in the community about mental health disorders but it is still far from enough. Abuse and discrimination still occur and cultural aspects are involved in explanations for the origin of mental health disorders. Providing information and education to the community about mental health have begun to change the level of knowledge and potentially will continue for the improvement of health and care. The integration of mental health into PHC can also contribute to the reduction of stigma for mental health disorders [31]. Culture, tradition and religion are aspects in Cambodia that may be both inhibitive and important for the improvement of mental health. A psychiatrist pointed out that patients could describe their mental health symptoms as physical symptoms, which might be a cultural manifestation important to be aware of. There has been described the need to provide mental health care services that are culturally-sensitive in order to increase in order to access and usage of the services [32]. Educating traditional healers in mental health has the potential to reach people in their own context and from there, start to improve the care, which has already been recognised in other studies [33, 34].

### Monitoring and evaluation

The lack of monitoring and evaluation for mental health has contributed to the inadequate data available about mental health disorders and care. To improve the knowledge of and work with mental health, it is of high interest to have adequate documentation both in the community and at higher levels. A better monitoring of mental health outcomes and a proper evaluation of mental care interventions can contribute to place mental health higher on the national agenda.

### Key stakeholders

The results for this section were limited due to the low response, although several key stakeholders were mentioned by two respondents. Another study reported that policy makers, donors, mental health specialists, the media and universities were the most powerful and most supportive actors for scaling up mental health care in the respective PRIME countries [35].

### Cross-country challenges for the integration of mental health into PHC

It has been argued that integration may be the only feasible option to address mental health problems in the context of a weak health system. Furthermore, this can contribute to health systems’ improvements generally and the overall well-being of the population. In the work of integrating mental health into PHC, it is important to assess the goals, functions and resources for the integration programmes and to assign responsibilities and establish a monitoring mechanism to reduce the risk of failure [36].

The literature provides different examples of this integration. A study from Zambia highlighted how integrating mental health services into PHC was critical for improving the mental health of the population in the country [31]. In India it was found that primary care doctors described integration programmes of mental health into PHC as valuable and that they became more involved in the integration due to observed benefits of patients’ clinical outcomes and positive feedback from the patients [37]. Interviews with health care workers in Lvea Em District also show support of the integration of mental health services into PHC and most of them were willing to personally deliver mental health care [38].

In 2011 the United States National Institute of Mental Health (NIMH) identified the 25 grand challenges in global mental health where to ‘strengthen the mental health component in the training of all health-care personnel’ and to ‘integrate screening and core package of services into routine PHC’ were two of the top challenges [39]. Most of the challenges seem to be relevant even for Cambodia where training in mental health and resources are also highlighted in this study.

### Comparison to the PRIME study

The study of Lvea Em District and Cambodia can be compared with the PRIME study conducted in five districts in India, Nepal, South Africa, Ethiopia and Uganda by Hanlon et al. [25]. The lack of specialists in mental health, pre-service training, limited availability of psychotropic drugs, screening tools and the absence of guidelines for interventions and treatment protocols for mental health disorders are commonly shared factors with our study. Lvea Em District and the PRIME districts need more information about treatment coverage for mental health disorders as an essential part of the process of scaling up mental health into PHC. The absence of health system structures supporting mental health care are the same between the countries and is crucial for the development of mental health care both in the districts and in the countries overall.

Common to the districts were the limited availability to provide care for patients with mental health disorders and the limited referral to specialist mental health services. However, there seemed to be a more developed referral system in the other districts compared to Lvea Em. There was more clarity about the permission for prescriptions of psychotropic medications in the PRIME districts compared to Lvea Em District.

## Strengths and limitations

A strength of this study is that data were collected mainly from first-hand sources from different levels, including personnel in the PHC to national level, which contributes to more points of view on the same issues. The research was based on a validated method with the PRIME-tool from PRIME, making the results more reliable.

One of the reasons for the selection of Lvea Em District was the already established collaboration between the district health authorities and CCAMH. The assumption was that the established contact had led to more involvement from the participants in the district and encouraged the interest in mental health and care. The circumstances of the collaboration might have contributed to a certain response bias since the respondents might have felt influenced by the project members’ background (as academic and as the interviewer was a Westerner) and possible expectations of the project.

Due to the lack of time, the questionnaire was pre-tested by only one person and the questionnaire was not back-translated before being used in the district. However, two persons were involved in the interpretation, reducing the risk of incorrect translations. The lack of training in mental health may also have contributed to the difficulties in completing the questionnaire for the participants. Some of the questions were not answered by all participants, which has led to certain inaccuracy in some of the results. The uncertainties in the answers (observed at all levels) should not only be seen as a limitation in the study, but as an indicator of the insufficient system available today for mental health and mental health care. The language barrier also probably affected the results in the way that the meaning of words and expressions can be different between the languages involved (Swedish, English and Khmer).

A final limitation of this study was that no interviews were held with mental health service users and their families; this could have contributed to expand the different aspects of the mental health situation. This issue may be of great interest for any future project similar to this one.

## Conclusions

There are both similarities and differences between Lvea Em District and Cambodia as a whole but the overall conclusion is that the resources for mental health care are far below enough both in the country and in the district. Neither the government nor non-governmental organizations (NGOs), with the exception of a few, have really focused on mental health in Cambodia. The result is that the public mental health services are of limited quality, there is an absence of multi-sectoral approaches to mental health care and there is a lack of unified leadership and vision.

This study highlights challenges and opportunities for the work of integrating mental health into PHC together with the need for a comprehensive intervention and scaling up in the area of mental health and health care in the country.

## Additional file

**Additional file 1.** Adapted PRIME situation analysis tool.
