# Supplementary material for: Mental health in primary health care in a rural district of Cambodia: a situational analysis
Source: Int J Ment Health Syst. 2018 Jan 24;12:7. doi: 10.1186/s13033-018-0185-3 (PMC5782361; doi:10.1186/s13033-018-0185-3)
Supplement: Supplementary file 1 — Additional file 1. Adapted PRIME situation analysis tool. [file 13033_2018_185_MOESM1_ESM.pdf]

PRIME
កម្មវិធីសម្រាប់ការថែទាំសុខភាពផ្លូវចិត្តឲ្យបានប្រសើរឡើង

PRIME
Programme for improving mental health care

ឧបករណ៍ការវិភាគស្ថានភាព
អភិវឌ្ឍន៍ដោយកម្មវិធីសម្រាប់ការថែទាំសុខភាពផ្លូវចិត្តឲ្យបានប្រសើរឡើង

Situational Analysis Tool
Developed by the Programme for Improving Mental Health Care

កំរងសំនួរសម្រាប់មណ្ឌលថែទាំសុខភាពបឋមនីមួយៗ នៅក្នុងស្រុកល្វាឯម

Questionnaire for each Primary Health Care Center in Lvea Em District

ទម្រង់សម្រាប់ការសុំអនុញ្ញាត

ទម្រង់សំនួរនេះ គឺត្រូវបានប្រើប្រាស់សម្រាប់គម្រោងសិក្សាពីស្ថានភាពបច្ចុប្បន្ននៃសេវាកម្ម សុខភាពផ្លូវចិត្ត នៅក្នុងស្រុកល្វាឯម។ គោលបំណងនៃគម្រោងនេះ គឺធ្វើការស្រាវជ្រាវយល់ជាបឋម/ មូលដ្ឋានសម្រាប់ការថែទាំសុខភាពផ្លូវចិត្តនៅក្នុងការថែទាំបឋម,ពត៌មាននេះអាចនឹងជួយ នៅក្នុងការដាក់បញ្ចូលការថែទាំសុខភាពផ្លូវចិត្ត នៅក្នុងការថែទាំសុខភាពបឋមនៅក្នុងប្រទេសកម្ពុជា។ គម្រោងនេះត្រូវបានទទួលស្គាល់ដោយមានការអនុញ្ញាតិបញ្ជាក់ពីក្រុមសីលធម៌រួចហើយ។

ទិន្នន័យនឹងត្រូវបានប្រមូលពីបុគ្គលិកធ្វើការនៅមណ្ឌលថែទាំសុខភាពបឋម នៅក្នុងស្រុក ល្វាឯម និងពីបុគ្គលិកមកពីថ្នាក់ខេត្ត និងថ្នាក់ជាតិ។ ការចូលរួមក្នុងការសិក្សាស្រាវជ្រាវនេះ គឺស្រេចលើចិត្ត។ ឈ្មោះរបស់អ្នក គឺមិនបានប្រើប្រាស់ នៅក្នុងរបាយការណ៍នោះទេ។

យើងខ្ញុំសូមថ្លែងអំណរគុណសម្រាប់ការចូលរួមរបស់លោកលោកស្រី ក្នុងការបំពេញកម្រង សំនួរ-នេះ។ ប្រសិនបើអ្នកចង់ដឹងពត៌មានបន្ថែម ឬមានសំណួរណាមួយ លោកអ្នក អាចទាក់ទងមកយើងខ្ញុំបាន។

ដោយក្តីគោរពរាប់អាន!

សូហ្វីយ៉ាង អូឡូហ្វស៊ិន (sool0014@student.umu.se)
សិស្សវិជ្ជសាស្រ្ត
សាកលវិទ្យាល័យ អ៊ូមៀ
ប្រទេសស៊ុយអែត

មីហ្កែល សាន សេបាសជៀន
វិទ្យាស្ថានសិក្សារោគរាតត្បាត និងសុខភាពសកល
សាកលវិទ្យាល័យអ៊ូមៀ
ប្រទេសស៊ុយអែត

ប៊ូមីកុមារ ជេហ្គាណាថាន់
មជ្ឈមណ្ឌលសុខភាពផ្លូវចិត្តកុមារ និងយុវវ័យ
មន្ទីរពេទ្យបង្អែក ជ័យជំនះ
ខេត្តកណ្តាល កម្ពុជា
ទូរស័ព្ទ: 023 983 348, 012 482 854
bhoomikumar.jegannathan@caritascambodia.org

ផ្នែកទីIII: គ្របដណ្តប់ការព្យាបាលសុខភាពផ្លូវចិត្ត<br>SECTION III: Mental health treatment coverage				
		ស្ថានភាពដំបូង Baseline situation		
		មណ្ឌលថែទាំសុខភាពបឋម Primary Health Care Center		
2.	ចំនួនអ្នកជំងឺដែលមករកសេវាកាលពីឆ្នាំមុន<br>NUMBER OF PEOPLE WITH THE DISORDER IN THE CONTACT WITH SERVICES IN THE LAST YEAR			
2.1	ជំងឺធ្លាក់ទឹកចិត្ត Depression			
2.2	ជំងឺថប់បារម្ភ Anxiety			
2.3	បញ្ហាគេង Sleep problem			
2.4	បញ្ហាសុរា Alcohol problem			
2.5	វិបល្លាស វិកលចរិកស្រួចស្រាវ<br>Acute psychotic disorder			
2.6	វិបល្លាស វិកលចរិក រ៉ាំរ៉ៃ Chronic psychotic disorder			
2.7	ជំងឺប្រកាច់ Epilepsy			
2.8	កុមារដែលមានការអភិវឌ្ឍន៍យឺតយ៉ាវ<br>Children with development delay			
2.9	កុមារដែលមានបញ្ហាអាក្បកិរិយា<br>Children with behaviour problems			
3.	គ្របដណ្តប់ការព្យាបាល<br>TREATMENT COVERAGE<br>ចំនួនអ្នកជំងឺដែលមករកសេវា/ប៉ាន់ប្រមាណអត្រាភាគរយ Number of people with disorder in contact with services / estimated prevalence			
3.1	ជំងឺធ្លាក់ទឹកចិត្ត Depression			
3.2	ជំងឺថប់បារម្ភ Anxiety			
3.3	បញ្ហាគេង Sleep problem			
3.4	បញ្ហាសុរា Alcohol problem			
3.5	វិបល្លាស វិកលចរិកស្រួចស្រាវ<br>Acute psychotic disorder			
3.6	វិបល្លាស វិកលចរិក រ៉ាំរ៉ៃ Chronic psychotic disorder			
3.7	ជំងឺប្រកាច់ Epilepsy			
3.8	កុមារដែលមានការអភិវឌ្ឍន៍យឺតយ៉ាវ<br>Children with development delay			
3.9	កុមារដែលមានបញ្ហាអាកប្បកិរិយា<br>Children with behaviour problems			

ផ្នែកទី IV: សេវាសុខភាពនៅកំរិតស្រុក
SECTION IV: DISTRICT LEVEL HEALTH SERVICES

		ស្ថានភាពដំបូង **Baseline situation**		
**1**	រចនាសម្ព័ន្ធរដ្ឋបាលសម្រាប់សេវាកម្មសុខភាព **ADMINISTRATIVE STRUCTURES FOR HEALTH SERVICE**			
**1.2**	**តើអ្នកប្រើសេវាកម្ម បានចូលរួមចំណែកកំរិតណាក្នុងដំនើរការ/អភិវឌ្ឍន៍សេវាកម្ម?** How much involvement of service users in service running / development?	ការចូលរួមចំណែកពេញលេញ **Full involvment**	ការចូលរួមចំណែកខ្លះៗ **Somewhat**	មិនបានចូលរួមចំណែក **Not at all**
**2**	ធនធានមនុស្សដែលមាន **AVAILABLE HUMAN RESOURCES**	ផ្នែកសេវាសាធារណៈ: **Public sector**		
**2.1**	បុគ្គលិកសុខភាពទូទៅនៅឯមណ្ឌលថៃទាំសុខភាព **General health workers at the care center**	មិនបំពេញមុខការ **Unfilled posts**	មានបំពេញមុខការ **Filled posts**	
	ឆ្មប Midwives			
	គិលានុប្បដ្ឋាកមធ្យម Degree nurses			
	គិលានុប្បដ្ឋាកបឋម Diploma nurses			
	ឱសថការី Pharmacists			
	ភ្នាក់ងារសុខភាពតាមសហគមន៍ Community health agents			
	បុគ្គលិកកិច្ចសន្យាសុខភាព (បុគ្គលិកសុខភាពសហគមន៍ទទួលបានប្រាក់កំរៃ) Health extension workers (paid community health workers)			
	បុគ្គលិកគាំទ្រសេវាពន្យាកំណើត Family planning support workers			
	បុគ្គលិកគ្រប់គ្រងករណីជំងឺអេដស៍ Case managers (ART)			
	បុគ្គលិកបរិដ្ឋាន Environmental health			
	បុគ្គលិកសុខភាពទូទៅ Lay health workers			
	ឆ្មបបុរាណ (បានបណ្តុះបណ្តាល) Traditional birth attendants (TBAs) (trained)			
	ឆ្មបបុរាណ (មិនបានបណ្តុះបណ្តាល) TBAs (untrained)			
	ផ្សេងៗទៀត Other			
**2.2**	**បុគ្គលិកឯកទេសសុខភាពផ្លូវចិត្ត/ប្រព័ន្ធប្រសាទ/ការប្រើប្រាស់សារធាតុញៀន** **Specialist mental health / neurology / substance misuse workers**			
	តើលោកអ្នកមានបុគ្គលិកសម្រាប់ព្យាបាលអ្នកជំងឺផ្លូវចិត្តដែរឬទេ? (ឧ: វេជ្ជបណ្ឌិតជំនាញប្រព័ន្ធប្រសាទ, វេជ្ជបណ្ឌិតឯកទេសវិកលវិទ្យា, គិលានុប្បដ្ឋាកឯកទេសវិកលវិទ្យា, អ្នកចិត្តវិទ្យា, អ្នកចិត្តវិទ្យា) Do you have any workers for mental health disorders (for example neurologist, psychiatrist, psychiatric nurse, psychologist)?	**បាទ/ចាស Yes**	**ទេ No**	
	បើមាន តើបុគ្គលិកជំនាញប្រភេទណា? If any, what kind of workers?			

3	**ការថែទាំអ្នកជំងឺស្នាក់នៅ**<br>**AVAILABLE IN-PATIENT CARE**			
3.1	**ការថែទាំអ្នកជំងឺស្នាក់ សម្រាប់ការថែទាំសុខភាពទូទៅ**<br>**Available in-patient care for general health care**	**មនុស្សចាស់**<br>**Adult**	**កុមារ**<br>**Children**	**សរុប**<br>**Overall**
	នៅឯមណ្ឌលសុខភាព At the health center			
	ប្រសិនបើគ្មាន តើកន្លែងណាដែលនៅជិតជាងគេ សម្រាប់អ្នកជំងឺស្នាក់?<br>If none, where is nearest general in-patient facility?			
	នៅឆ្ងាយប៉ុន្មាន? How far away?			
	តើមានផ្តល់សេវាបន្សាបសុរា នៅមណ្ឌលសុខភាពដែរឬទេ?<br>Is alcohol detoxification offered at the health center?	**បាទ/ចាស**<br>**Yes**	**ទេ**<br>**No**	
3.2	**ការថែទាំអ្នកជំងឺស្នាក់ សម្រាប់ការថែទាំសុខភាពផ្លូវចិត្ត**<br>**Available in-patient care for mental health care**	**មនុស្សចាស់**<br>**Adult**	**កុមារ**<br>**Children**	**សរុប**<br>**Overall**
	នៅឯមណ្ឌលសុខភាព At the health center			
	ប្រសិនបើគ្មាន តើកន្លែងណាដែលនៅជិតជាងគេ សម្រាប់អ្នកជំងឺស្នាក់?<br>If none, where is nearest mental health in-patient facility?			
	នៅឆ្ងាយប៉ុន្មាន? How far away?			
	តើកន្លែងណាដែលនៅជិតជាងគេ សម្រាប់អ្នកជំងឺញៀនសុរា ស្នាក់ព្យាបាល?<br>Where is the nearest specialist in-patient facility for alcohol abuse?			
4	**ការថែទាំសុខភាពបឋម**<br>**PRIMARY HEALTH CARE**			
4.2	**ជាទូទៅ តើប្រជាជនត្រូវធ្វើដំណើរប៉ុន្មានគីឡូម៉ែត្រ ដើម្បីទៅរកសេវានៅមណ្ឌលថែទាំសុខភាព?**<br>**(គិតជា គីឡូម៉ែត្រ)**<br>**Typically, how far do people have to travel to access the primary health care center? (kilometer)**			
4.3	**មុខងារ និងគ្របដណ្តប់ប្រជាជន ដោយមណ្ឌលថែទាំសុខភាព Function and population covered by the primary health care center?**			
	មុខងារ? Function?			
	គ្របដណ្តប់ចំនួនប្រជាជនប៉ុន្មាន? Population covered?			
5	**ការថែទាំសុខភាពតាមសហគមន៍ COMMUNITY HEALTH CARE**			
5.1	**តើមានបុគ្គលិកសុខភាពតាមសហគមន៍ទទួលបានប្រាក់កំរៃដែរឬទេ?**<br>**Are there any paid community health workers?**			
	តើមានប៉ុន្មានគ្រួសារ ដែលពួកគាត់ផ្តល់សេវា?<br>How many families do they serve?			
	តើពួកគាត់មានតួនាទីអ្វីខ្លះ? What is their role?			

	តើពួកគាត់ជួបអ្នកជំងឺផ្លូវចិត្តដែរឬទេ?<br>Do they meet patients with mental disorder?	
	តើពួកគាត់មានការបណ្តុះបណ្តាលកំរិតអ្វីខ្លះ?<br>What level of training do they have?	
5.2	**តើមានបុគ្គលិកសុខភាពតាមសហគមន៍ដែលមិនទទួលបានប្រាក់កំរៃដែរឬទេ?<br>Are there any unpaid community health workers?**	
	តើមានប៉ុន្មានគ្រួសារ ដែលពួកគាត់ផ្តល់សេវា?<br>How many families do they serve?	
	តើពួកគាត់មានតួនាទីអ្វីខ្លះ? What is their role?	
	តើពួកគាត់ជួបអ្នកជំងឺផ្លូវចិត្តដែរឬទេ?<br>Do they meet patients with mental disorder?	
	តើពួកគាត់មានការបណ្តុះបណ្តាលកំរិតអ្វីខ្លះ?<br>What level of training do they have?	
5.3	**សេវាកម្មចុះផ្ទាល់<br>Outreach services**	
	តើមានការចុះផ្ទាល់ដល់សហគមន៍ដើម្បីឲ្យអ្នកជំងឺផ្លូវចិត្តត្រលប់មករកការថែទាំវិញដែរឬទេ ប្រសិនបើពួកគាត់ឈប់មកតាមដាន?<br>Any scope for community outreach to get patients with mental disorders back into care if they get lost to follow up?	
5.4	តើមានអ្នកជំងឺផ្លូវចិត្តដែលអត់មានផ្ទះសម្បែង នៅម្តុំមណ្ឌលសុខភាពដែរឬទេ?<br>**Any homeless mentally ill persons in the up-take area of the health center?**	
6	**អ្នកឯកទេសុខភាពផ្លូវចិត្ត/ការថែទាំពិនិត្យជំងឺប្រព័ន្ធប្រសាទ<br>SPECIALIST MENTAL HEALTH / NEUROLOGICAL OUT-PATIENT CARE**	
6.1	តើកន្លែងដែលនៅជិតបំផុត មានចម្ងាយប៉ុន្មាន? តើទាមទាររយៈពេលប៉ុន្មាន? (រយៈពេលធ្វើដំណើរ, បើកសេវាពីម៉ោងណា)?<br>**How far away is the nearest facility? How accessible (time taken, opening hours)?**	
6.2	**តើមានបុគ្គលិកណាខ្លះ ដែលនៅជិតបំផុត?<br>Who staffs the nearest facility?**	
6.3	**ពិពណ៌នាពីសេវាកម្មដែលមាននៅជិតបំផុត?**<br>សេវាសម្រាប់អ្នកជំងឺស្នាក់, ពិនិត្យក្រៅ, ការព្យាបាលផ្លូវចិត្ត, ការស្តារលទ្ធភាពពលកម្មឡើងវិញ, ចុះផ្ទាល់<br>**Describe services available in nearest facility?**<br>In-patient, out-patient, psychological therapies, rehabilitation, outreach.	
6.4	**តើបុគ្គលិកជាវេជ្ជបណ្ឌិតឯកទេសវិកលវិទ្យា ដែលនៅជិតបំផុតមានចម្ងាយប៉ុន្មាន? How far away is nearest facility staffed by psychiatrist?**	
6.5	**តើសេវាកម្មពិនិត្យ អ្នកជំងឺញៀនសុរា ដែលនៅជិតបំផុតមានចម្ងាយប៉ុន្មាន? How far away is nearest specialist out-patient alcohol service?**	

7	សុខភាពផ្លូវចិត្តក្នុងការថែទាំសុខភាពមតា នៅក្នុងមណ្ឌលសុខភាព<br>**MENTAL HEALTH IN MATERNAL HEALTH CARE, AT THE CARE CENTER**			
7.1	ពិពណ៌នាពីសភាពទូទៅ នៃសេវាកម្មសុខភាពមាតា<br>**Describe nature of maternal health services**			
7.3	សេវាកម្មសម្រាលកូន, ចម្ងាយ និងបុគ្គលិក<br>**Delivery services, distances and staffing**			
7.4	តើមានការថែទាំតាមផ្ទះដែរឬទេ?<br>នរណាជាអ្នកផ្តល់សេវាកម្ម? សម្រាប់ស្រ្តីទាំងអស់រឺ?<br>**Any home-based care? Who provides this? For all women?**			
7.5	ពិពណ៌នាពីការថែទាំក្រោយសម្រាល<br>(ទីកន្លែង/ធ្វើតាមគោលការណ៍ ពិធីការផ្សេងៗ)<br>**Describe postnatal care**<br>Location/protocols followed			
7.6	តើមានប្រព័ន្ធហិរញ្ញវត្ថុអ្វីខ្លះ សម្រាប់ការថែទាំសុខភាពមាតា? (មូលនិធិសមធម៌សុខភាព)<br>**What is financing system for maternal health care? (Health Equity fund)**			
7.7	តើមានកត្តាផ្សេងៗ ដែលពាក់ព័ន្ធនឹងការផ្តល់ការថែទាំសុខភាពមាតាដែរឬទេ?<br>**Any other relevant factors in relation to delivery of maternal health care?**			
7.8	តើមានការថែទាំសុខភាពផ្លូវចិត្តអ្វីខ្លះ នៅក្នុងស្រុកនេះ សម្រាប់ស្រ្តីដែលជិតសម្រាលកូន?<br>**What mental health care is provided in the district for perinatal women?**			
7.9	ប្រសិនបើគ្មាន តើកន្លែងណាដែលនៅជិតបំផុត សម្រាប់ស្រ្តី អាចទទួលបានការថែទាំសុខភាពផ្លូវចិត្តដែលជិតសម្រាលកូន?<br>**If none, where is the nearest place that a women can obtain mental health care during perinatal period?**			
7.10	តើស្រ្តីប្រឈមមុខនឹងបញ្ហា/ឧបសគ្គអ្វីខ្លះកំលុងពេលមានផ្ទៃពោះ/ក្រោយពេលសម្រាល ដើម្បីទទួលបានការថែទាំសុខភាពផ្លូវចិត្ត?<br>**What barriers do women face in pregnancy / the postnatal period to accessing mental health care?**			
8	ការថែទាំសុខភាពអេដស៍ នៅមណ្ឌលថែទាំសុខភាព<br>**HIV HEALTH CARE AT THE CARE CENTER**			
	ការថែទាំអ្នកជំងឺអេដស៍ទូទៅ<br>**General HIV care**	ស្រ្តី<br>**Female**	បុរស<br>**Male**	សរុប<br>**Overall**
8.1	ចំនួនអ្នកជំងឺអេដស៍ដែលប្រើប្រាស់សេវាកម្មក្នុង១ខែ<br>**Number of people using HIV services per month**			

	**ពិពណ៌នាពីសភាពទូទៅនៃសេវាកម្មអេដស៍ នៅឯមណ្ឌលថែទាំសុខភាព (ទីកន្លែង, បុគ្គលិក, សេវាកម្មដែលផ្តល់អ្នកជំងឺ)**<br>Describe nature of HIV services at the care center (location,staffing, service offered)	
**8.2**	**ការថែទាំសុខភាពផ្លូវចិត្ត ក្នុងសេវាកម្មអេដស៍**<br>**Mental health care in HIV services**	
	តើការថែទាំសុខភាពផ្លូវចិត្តអ្វីខ្លះ ដែលបានផ្តល់នៅមណ្ឌលសុខភាពសម្រាប់អ្នកជំងឺអេដស៍?<br>What mental health care is provided at the care center for PLWHA (person living with HIV/AIDS)?	
	ប្រសិនបើគ្មាន តើកន្លែងណាដែលនៅជិតបំផុតសម្រាប់អ្នកជំងឺអេដស៍ទទួលបានការថែទាំសុខភាពផ្លូវចិត្ត?<br>If none, where is the nearest place that a PLWHA can obtain mental health care?	
	តើមានឧបសគ្គអ្វីខ្លះដែលអ្នកជំងឺអេដស៍ជួបប្រទះ ក្នុងការទទួលបានការថែទាំសុខភាពផ្លូវចិត្ត?<br>What barriers do PLWHA face in accessing mental health care?	
**10**	**ការបណ្តុះបណ្តាលសុខភាពផ្លូវចិត្តនៅក្នុងមណ្ឌលសុខភាពបឋម**<br>**MENTAL HEALTH TRAINING IN PHC**	
**10.1**	**ការបណ្តុះបណ្តាលមុនពេលផ្តល់សេវាកម្ម**<br>**Pre-service training**	
	តើមានការបណ្តុះបណ្តាលមុនពេលផ្តល់សេវាកម្មអ្វីខ្លះពីសុខភាពផ្លូវចិត្តដល់បុគ្គលិកមណ្ឌលសុខភាពបឋម? (បញ្ជាក់ពីកំរិតសញ្ញាប័ត្របុគ្គលិក រយៈពេល និងការបណ្តុះបណ្តាលបែងចែកទ្រឹស្តី និងការអនុវត្តន៍)<br>What pre-service training is provided in mental health for PHC workers? (specify grade of worker, duration, separating theoretical and practical training)?	
**10.2**	**ការបណ្តុះបណ្តាលពេលផ្តល់សេវាកម្ម**<br>**In-service training**	
	តើគិលានុប្បដ្ឋាក មណ្ឌលសុខភាពប៉ុន្មានភាគរយ បានទទួលការបណ្តុះបណ្តាលយ៉ាងហោចណាស់រយៈពេល ២ថ្ងៃ ពីសុខភាពផ្លូវចិត្ត កាលពីឆ្នាំមុន?<br>What % of PHC nurses received at least 2 days of refresher training in mental health in last 1 year?	
	ពិពណ៌នាពីសភាពទូទៅនៃការបណ្តុះបណ្តាល, រយៈពេល, ការបណ្តុះបណ្តាលធ្វើឡើងដោយនរណា, ការបណ្តុះបណ្តាលផ្តោតទៅលើអ្វី?<br>Describe nature of any training, duration, who conducted the training, focus of training	

11	ការព្យាបាលសុខភាពផ្លូវចិត្តនៅក្នុងមណ្ឌលសុខភាពបឋម<br>**MENTAL HEALTH TREATMENTS IN PHC**			
11.3	**ថ្នាំព្យាបាលជំងឺផ្លូវចិត្ត ដែលមាននៅមណ្ឌលសុខភាព**<br>**Psychotropic medication availability at the health centre**	**ថ្នាំដែលមាន**<br>**Medication available**	**ផ្គត់ផ្គង់ទៀងទាត់**<br>**Regularity of supply**	**បន្តការផ្គត់ផ្គង់**<br>**Continuity of supply**
	ថ្នាំលេបប្រឆាំងជំងឺវិកលចរិក Antipsychotics (po)			
	ថ្នាំចាក់ប្រឆាំងជំងឺវិកលចរិក Antipsychotic depot			
	ថ្នាំប្រឆាំងជំងឺធ្លាក់ទឹកចិត្ត Antidepressants			
	ថ្នាំ Anxiolytics			
	ថ្នាំសម្រួលអារម្មណ៍ Mood-stabilisers			
	ថ្នាំប្រឆាំងជំងឺប្រកាច់ Antiepileptics			
	ថ្នាំផ្សេងៗទៀត Other			
11.5	**យន្តការសម្រាប់ការផ្តល់ហិរញ្ញប្បទានថ្នាំ ប្រឆាំងជំងឺវិកលចរិក** (ឧ: បង់លុយ, ធានារ៉ាប់រង, អត់បង់លុយ, បង់សងជំនួស)<br>**Mechanisms for psychotropic medication financing** (e.g. out-of-pocket, insurance, waiver schemes, reimbursement)			
11.6	**មិនគិតថ្លៃថ្នាំទាំងស្រុង** តើប្រជាជនប៉ុន្មានភាគរយ នៅតំបន់នេះ បានទទួលថ្នាំប្រឆាំងជំងឺវិកលចរិក ដោយឥតគិតថ្លៃ (យ៉ាងហោចណាស់ ៨០% នៃតម្លៃដែលត្រូវគិត)?<br>**Free medication** Overall, what % of population in the up-take area have access to free psychotropic medication (at least 80% of costs covered)?			
11.8	**ការព្យាបាលចិត្តសង្គម**<br>**Psychosocial therapies**			
	តើមានការអន្តរាគមន៍ចិត្តសង្គមនៅក្នុងមណ្ឌលសុខភាពដែរឬទេ? (ឧ: ការប្រឹក្សា គាំទ្រយោបល់, ការអន្តរាគមន៍សាមញ្ញសម្រាប់អ្នកញៀនសុរា, ការព្យាបាលចិត្តសង្គមផ្សេងៗទៀត)<br>Are any psychosocial interventions available at the care center (for example supportive counselling, brief interventions for alcohol, other psychosocial therapy)?	**បាទ/ចាស**<br>**Yes**	**ទេ**<br>**No**	
	ប្រសិនបើមាន តើការព្យាបាលប្រភេទអ្វីខ្លះ?<br>If any, what kind of therapies?			
12	**មណ្ឌលសុខភាពបឋម/ការបញ្ជូនសេវាកម្មផ្លូវចិត្ត**<br>**PHC / MENTAL HEALTH SERVICE INTERFACE**			
12.1	ចំនួនអ្នកជំងឺដែលបានបញ្ជូនទៅរកអ្នកឯកទេសសេវាផ្លូវចិត្ត (ក្នុងបុគ្គលិក១នាក់/ខែ បញ្ជូនអ្នកជំងឺបានប៉ុន្មាននាក់?Number of patients referred for specialist mental health services (per PHC worker / month)			

**12.2**	**ទំនាក់ទំនងរវាងបុគ្គលិកមណ្ឌលសុខភាព និងអ្នកជំនាញសុខភាពផ្លូវចិត្តកាលពីឆ្នាំមុន** (ភាពញឹកញាប់ប៉ុន្មាន?) **Contact between PHC workers and mental health professionals in last year** (frequency)			
**12.3**	**យន្តការបញ្ជូនសម្រាប់សុខភាពផ្លូវចិត្ត, ចូរពិពណ៌នា?**<br>**Referral mechanisms for mental health, describe**			
	ដំណើរការបញ្ជូនជាផ្លូវការ<br>(ពីមណ្ឌលសុខភាព→ ការថែទាំកំរិតទី២ ទី៣)<br>Official referral procedures<br>(PHC -> secondary / tertiary care)			
	ដំណើរការបញ្ជូនជាផ្លូវការ<br>(ពីការថែទាំកំរិតទី២/ទី៣ →មណ្ឌលសុខភាពបឋម<br>Official referral procedures<br>(secondary / tertiary care -> PHC)			
**12.4**	យោបល់ពីប្រព័ន្ធបញ្ជូន (ជាទ្រឹស្តី/ការអនុវត្តន៍) ឧបសគ្គនានា<br>**Comments on referral systems (in theory / in practice). Obstacles.**			
**13**	**មណ្ឌលសុខភាពបឋម/ការបញ្ជូនទៅសហគមន៍**<br>**PHC / COMMUNITY INTERFACE**			
**13.1**	**ការទំនាក់ទំនងសហគមន៍ជាមួយមណ្ឌលសុខភាពបឋម**<br>ក្រុមស្ម័គ្រចិត្ត/អង្គការជំនឿផ្សេងៗ/គ្រូបុរាណ/ក្រុមគ្រួសារ ។ល។<br>តើសេវាមណ្ឌលសុខភាពមានទំនាក់ទំនងយ៉ាងដូចម្តេចជាមួយពួកគាត់?<br>**Community linkages with PHC**<br>Volunteers / faith-based organisations / traditional healers / family groups, etc.<br>How do PHC services link with them?			
**13.3**	**តើមណ្ឌលសុខភាពមានទំនាក់ទំនងជាមួយគ្រូខ្មែរបុរាណ យ៉ាងហោចណាស់១ដង/ក្នុង១ឆ្នាំដែរឬទេ?**<br>**Do the health center interact with traditional / religious healers at least once per year?**	**បាទ/ចាស**<br>**Yes**	**ទេ**<br>**No**	
	ប្រសិនបើមាន តើញឹកញាប់ប៉ុណ្ណា?<br>If Yes, how often?			
**14**	**ប្រព័ន្ធគាំទ្រការថែទាំសុខភាពផ្លូវចិត្តនៅក្នុងមណ្ឌលសុខភាព**<br>**SYSTEMS TO SUPPORT MENTAL HEALTH CARE IN PHC**			
**14.4**	**តើមានឧបករណ៍សម្រាប់កត់សម្គាល់/ពិនិត្យសុខភាពផ្លូវចិត្តដែរឬទេ?**<br>**Mental health detection/ screening tools?**			
**14.4a**	តើមានគោលការណ៍ក្នុងការវាយតម្លៃ និងការព្យាបាលសម្រាប់ការថែទាំសុខភាពផ្លូវចិត្តដែរឬទេ?<br>Any guidelines / assessment and treatment protocols for mental health care?			

14.4b	តើមានសៀវភៅណែនាំចេញជាផ្លូវការ ស្ដីពីជំងឺផ្លូវចិត្ត នៅក្នុងមណ្ឌលសុខភាពដែរឬទេ? Are officially approved training manuals on mental disorders available in the health center?	បាទ/ចាស Yes	ទេ No	
14.4c	តើលោកអ្នកស្គាល់កញ្ចប់សកម្មភាពអប្បបរិមា (MPA) ដែរឬទេ? Are you aware of MPA guidelines?	បាទ/ចាស Yes	ទេ No	
14.5	**តើមានវិធីសាស្ត្រណា កត់សម្គាល់អ្នកជំងឺដែលបោះបង់ការព្យាបាលដែរឬទេ?** ចំនុចដែលពាក់ព័ន្ធនឹងការអនុវត្តន៍សុភាពផ្លូវចិត្ត? ការចុះឈ្មោះអ្នកជំងឺតាមប្រព័ន្ធការណាត់ជួប? **Any way of detecting patients who drop out of care?** Case registers? Appointment systems?			
15	**ចំនុចដែលពាក់ព័ន្ធនឹងការអនុវត្តន៍សុខភាពផ្លូវចិត្តទៅក្នុងមណ្ឌលសុខភាព** **RELEVANT BACKGROUND TO SCALING UP MENTAL HEALTH INTO PHC**			
15.0	**តើលោកអ្នកស្គាល់គោលការណ៍អន្តរជាតិនៃ ចន្លោះសុខភាពផ្លូវចិត្ត (mhGAP-IG) ដែរឬទេ?** **Do you know what mhGAP-IG is?**	បាទ/ចាស Yes	ទេ No	
15.1	**តើអ្នកឯកទេសនៅមណ្ឌលសុខភាពមួយណា អាចអនុវត្តន៍គោលការណ៍អន្តរជាតិ (ចន្លោះសុខភាពផ្លូវចិត្ត (mhGAP-IG) នោះ?** **Which PHC professionals might deliver mhGAP-IG?**			
15.2	**តើមានទិន្នន័យ/ឯកសារបោះពុម្ព/របាយការណ៍ស្ដីពីបទពិសោធន៍ នៃការអនុវត្តន៍សុខភាពផ្លូវចិត្តនៅក្នុងមណ្ឌលសុខភាពដែរឬទេ?** **Any existing data / publications / reports on experience of implementing mental health into PHC?**			

**ផ្នែកទី** V: **សហគមន៍** **SECTION V: COMMUNITY**		
		**ស្ថានភាពដំបូង Baseline situation**
**1.**	កត្តាវប្បធម៌-សង្គម **SOCIOCULTURAL FACTORS**	
**1.1**	តើមានទំនោរស្វែងរកការជួយសម្រាប់ជំងឺផ្លូវចិត្ត នៅក្នុងតំបន់មណ្ឌលសុខភាពដែរឬទេ? **Is there a tendency for help-seeking for mental disorders in the up-take area of the health center?**	
**1.2**	តើមានវត្តអារាម/គ្រូបុរាណ នៅក្នុងតំបន់នេះឬទេ? **Prominent holy sites (wat) / traditional healers in the area?**	
**1.3**	តើប្រភេទបញ្ហាសុខភាពផ្លូវចិត្តអ្វីខ្លះ ដែលគ្រូខ្មែរ/បុរាណព្យាបាល? (និងដោយវិធីយ៉ាងដូចម្ដេចខ្លះ?) **What mental health conditions do traditional / religious healers treat? (and how?)**	

**1.5**	តាមការប៉ាន់ប្រមាណ តើមានអ្នកដែលមានបញ្ហាផ្លូវចិត្ត ប៉ុន្មាននាក់ បានពិនិត្យដោយគ្រូខ្មែរ/បុរាណ នៅក្នុងតំបន់នេះ? (ឧ: ក្នុង១ខែ ប៉ុន្មាននាក់?)<br>**Any estimates of how many persons with mental health problems are seen by traditional healers / religious healers in the up-take area?** e.g. in one month		
**1.6**	វប្បធម៌<br>**Culture**<br>តើសហគមន៍មានជំនឿអ្វីខ្លះ លើគំរូនៃវិបល្លាស/បញ្ហាផ្លូវចិត្ត?<br>What is known about community believes in models of mental disorders?		
**1.7**	ភាពអាម៉ាស់/ការរើសអើង<br>**Stigma / discrimination**<br>តើមានភាពអាម៉ាស់/ការរើសអើងអ្វីខ្លះលើអ្នកដែលមានវិបល្លាសផ្លូវចិត្ត នៅក្នុងសហគមន៍?<br>What is known about stigma or discrimination against mentally disordered persons within the community?		
**1.8**	ការរំលោភបំពាន<br>**Abuse**<br>តើមានការអនុវត្តន៍រំលោភបំពានអ្វីខ្លះ? (ឧ: ការដាក់ច្រវ៉ាក់, ការចងឃុំ ។ល។) What is known of abusive practices e.g. chaining, restraining etc.?		
**1.9**	បន្ទុកក្រុមគ្រួសារ<br>**Family burden**<br>តើមានបន្ទុក ការគាំទ្រអ្វីខ្លះសម្រាប់ក្រុមគ្រួសារ (សេដ្ឋកិច្ចសង្គម) ថែទាំអ្នកដែលមានវិបល្លាសផ្លូវចិត្ត?<br>What is known of the burden / support (socioeconomic) for families caring for a person with mental disorder?		
**2**	ផ្នែកមិនមែនសុខភាព-សកម្មភាពដែលជាប់ពាក់ព័ន្ធនឹងសុខភាពផ្លូវចិត្ត<br>**NON-HEALTH SECTOR ACTIVITIES RELEVANT TO MENTAL HEALTH**		
**2.1**	តើមានអង្គការណាមួយ គាំទ្រមណ្ឌលសុខភាពលោកអ្នកដែរឬទេ? **Are there any NGO support for your center?**	មាន<br>**Yes**	គ្មាន<br>**No**
**2.2**	តើមានកម្មវិធីគាំទ្រជីវភាព/ការកាត់បន្ថយភាពក្រីក្រ/ពង្រឹងសមត្ថភាពនៅក្នុងតំបន់នេះដែរឬទេ? (រាប់ទាំងស្ថាប័នរដ្ឋ, វិស័យឯកជន/អង្គការនានា) **Any livelihood / poverty alleviation / empowerment programmes running in the up-take area? (public / private sectors / NGOs)**	មាន<br>**Yes**	គ្មាន<br>**No**
**2.3**	ក្រុមសហគមន៍<br>**Community groups**<br>មានក្រុមជួយខ្លួនឯង/ក្រុមគាំទ្រ/ក្រុមគ្រួសារដែរឬទេ?<br>Self-help / support / family groups?	មាន<br>**Yes**	គ្មាន<br>**No**

**2.4**	**តើមានការគាំទ្រសង់ជាផ្ទះសម្បែងដែរឬទេ?** ឬជួយពាក់កណ្តាលសម្រាប់អ្នកដែលមានជំងឺផ្លូវចិត្តធ្ងន់ធ្ងរ និងរ៉ាំរ៉ៃ?<br>**Supported housing?** Or half-way houses for persons with severe and enduring mental illness?	**មាន**<br>**Yes**	**គ្មាន**<br>**No**
**2.5**	**ការស្តារលទ្ធភាពឡើងវិញ / ការជាឡើងវិញ / ការបញ្ចូលក្នុងសង្គម?**<br>**Rehabilitation / recovery / social inclusion?**<br>តើមានសកម្មភាពក្នុងសហគមន៍ ដើម្បីគាំទ្រការជាឡើងវិញ និងសមារហាកម្មឡើងវិញ សម្រាប់អ្នកដែលមានជំងឺផ្លូវចិត្តដែរឬទេ?<br>Any community activities to support the recovery and reintegration of persons who have experienced mental illness?	**មាន**<br>**Yes**	**គ្មាន**<br>**No**
**3.**	ការលើកកម្ពស់ / ការការពារ / ការផ្សព្វផ្សាយបង្កើនការយល់ដឹង<br>**PROMOTION / PREVENTION / AWARENESS-RAISING**		
**3.1**	តើមានសកម្មភាពផ្សព្វផ្សាយឲ្យមានការយល់ដឹង/ប្រឆាំងភាពអាម៉ាស់ នៅក្នុងសហគមន៍ដែរឬទេ? ធ្វើឡើងដោយអ្នកណា?, នៅទីកន្លែងណា?, ញឹកញាប់ប៉ុណ្ណា?<br>**Any community awareness-raising / anti-stigma activities?**<br>Who, where, how often?	**មាន**<br>**Yes**	**គ្មាន**<br>**No**
**3.2**	តើមានសកម្មភាពការលើកកម្ពស់សុខភាពផ្លូវចិត្ត/ការការពារវិបល្លាសផ្លូវចិត្ត នៅក្នុងតំបន់នេះដែរឬទេ? ធ្វើឡើងដោយអ្នកណា? នៅទីកន្លែងណា? ញឹកញាប់ប៉ុណ្ណា?<br>**Any mental health promotion / mental disorder prevention activities within the area?**<br>Who, where, how often?	**មាន**<br>**Yes**	**គ្មាន**<br>**No**

សូមអរគុណសម្រាប់បំពេញនូវកំរងសំណួរនេះ!

(Thank you!)
